# *Mycobacterium tuberculosis* Transcriptional Adaptation, Growth Arrest and Dormancy Phenotype Development Is Triggered by Vitamin C

**DOI:** 10.1371/journal.pone.0010860

**Published:** 2010-05-27

**Authors:** Neetu Kumra Taneja, Sakshi Dhingra, Aditya Mittal, Mohit Naresh, Jaya Sivaswami Tyagi

**Affiliations:** 1 Department of Biotechnology, All India Institute of Medical Sciences, New Delhi, India; 2 School of Biological Sciences, Indian Institute of Technology Delhi, New Delhi, India; 3 Department of Biochemical Engineering and Biotechnology, Indian Institute of Technology Delhi, New Delhi, India; University of Hyderabad, India

## Abstract

**Background:**

Tubercle bacilli are thought to persist in a dormant state during latent tuberculosis (TB) infection. Although little is known about the host factors that induce and maintain *Mycobacterium tuberculosis* (*M. tb*) within latent lesions, O_2_ depletion, nutrient limitation and acidification are some of the stresses implicated in bacterial dormancy development/growth arrest. Adaptation to hypoxia and exposure to NO/CO is implemented through the DevRS/DosT two-component system which induces the dormancy regulon.

**Methodology/Principal Findings:**

Here we show that vitamin C (ascorbic acid/AA) can serve as an additional signal to induce the DevR regulon. Physiological levels of AA scavenge O_2_ and rapidly induce the DevR regulon at an estimated O_2_ saturation of <30%. The kinetics and magnitude of the response suggests an initial involvement of DosT and a sustained DevS-mediated response during bacterial adaptation to increasing hypoxia. In addition to inducing DevR regulon mechanisms, vitamin C induces the expression of selected genes previously shown to be responsive to low pH and oxidative stress, triggers bacterial growth arrest and promotes dormancy phenotype development in *M. tb* grown in axenic culture and intracellularly in THP-1 cells.

**Conclusions/Significance:**

Vitamin C mimics multiple intracellular stresses and has wide-ranging regulatory effects on gene expression and physiology of *M. tb* which leads to growth arrest and a ‘dormant’ drug-tolerant phenotype, but in a manner independent of the DevRS/DosT sytem. The ‘AA-dormancy infection model’ offers a potential alternative to other models of non-replicating persistence of *M. tb* and may be useful for investigating host-‘dormant’ *M. tb* interactions. Our findings offer a new perspective on the role of nutritional factors in TB and suggest a possible role for vitamin C in TB.

## Introduction

Tuberculosis (TB) is characterized by persistence of tubercle bacilli. There is an urgent need to understand the mechanisms underlying bacterial persistence and intracellular survival in order to devise means to control active disease. The intracellular environment exposes *M. tb* to multiple stresses that include, among others, hypoxic, nutrient limiting, oxidative, nitrosative and acidic conditions [1]–[3]. In vitro models of dormancy have identified hypoxia, nitric oxide (NO) and nutrient starvation as dormancy inducing signals [4]–[6]. Extensive transcriptional changes are triggered during hypoxia through the DevR response regulator in *M. tb* [7]. The crucial role of DevR in adaptation can be deduced from the finding that hypoxic viability is severely compromised in a *devR* mutant [8] or in wild type organisms exposed to a DevR inhibitor [9]. Interestingly, the defect is not as severe in a *devS* mutant of *M. bovis* BCG [8] suggesting that a homologous sensor kinase DosT/Rv2027c [10]–[12] may substitute for DevS. Similarities in phosphorylation and phosphotransfer properties and the presence of two potential signal-sensing GAF domains in DevS and DosT make them functionally analogous proteins and suggest that they could be involved in processing similar signals [11], [12]. Both DevS and DosT bind heme in their N-terminal GAF domain [13]–[17] and both proteins are enzymatically active kinases in the deoxy-Fe^2+^ state and inactive in the O_2_-bound state. NO and CO may serve as high affinity antagonists that readily replace O_2_ at the heme to generate the active kinase species [14], [16], [18], [19]. Interestingly, full induction of the DevR target gene, *hspX*, depends on both DevS and DosT [11].

The DevR regulon is induced under a variety of conditions including standing/centrifugation of cultures (both of which generate local hypoxia), exposure to NO, CO, ethanol, H_2_O_2_ and early during infection of macrophages, dendritic cells, mice and guinea pigs [3], [5], [18]–[24]. Furthermore, expression of *devS*, but not *dosT*, is hypoxia-inducible and the activities of the truncated kinases are modulated by cations, suggesting that the DevR regulon could respond to additional environmental cues [12]. In order to decipher the response of DevS/DosT to various cations, vitamin C (L-ascorbic acid or AA) was added to *M. tb* cultures to maintain iron in the reduced state. This led us to the discovery that AA rapidly induces the DevR regulon under aerobic conditions.

Vitamin C is a critical dietary nutrient in humans and involved in a number of vital cellular functions including collagen biosynthesis, facilitating iron transport and being a very important physiological antioxidant [25]. These activities of vitamin C affect a wide range of biological processes [25]. Several studies have described the beneficial effect of orally administered vitamin C in treating human and experimental TB [26], [27], while others have reported the occurrence of vitamin C deficiency in TB patients [28]. The outcome of a TB infection depends on an intricate and dynamic interplay between the host and the TB pathogen. The action of vitamin C in infectious disease is generally attributed to host protection from oxidative damage by ROIs and RNIs generated to kill intracellular bacteria [29] and effects on host immunity [30]. These properties are likely enabled by the ability of immune cells to concentrate AA to millimolar levels intracellularly [31]–[34]. In view of the pleiotropic effects of vitamin C on the host [25], [29], [30] and the serendipitous discovery of DevR regulon induction in the TB pathogen upon exposure to vitamin C, we further examined the effect of this vitamin on *M. tb* gene expression and physiology. We find that DosT and DevS mediate a rapid and sustained induction of the DevR regulon, respectively, in response to hypoxia achieved through the O_2_-scavenging and reducing property of AA. AA is shown to mediate the induction of some low pH and oxidative stress response genes. In this work, we also show that it promotes bacterial growth arrest and dormancy development in *M. tb* grown in axenic cultures and intracellularly in THP-1 cells. We discuss the significance of our findings for host-dormant *M. tb* interactions and TB control.

## Results

### Ascorbic acid mediates induction of the DevR dormancy regulon

**Figure 1:**
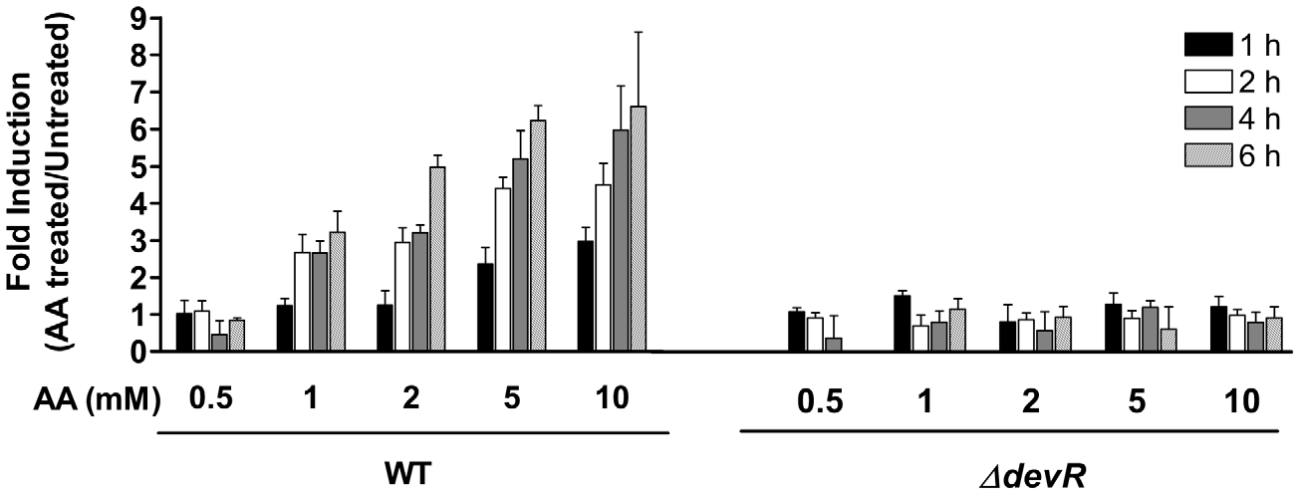
Induction of *Rv3134c* promoter activity in *M. tuberculosis*. The induction of the *Rv3134c* promoter (in plasmid p3134c-1) was assessed by measuring GFP fluorescence in 0–6 hours standing *M. tb* cultures. Fold induction of promoter activity in AA-exposed cultures is expressed with respect to untreated cultures, both under standing conditions. Results are expressed as mean ± SD of 3 independent experiments.

The effect of various cations including iron on *M. tb* dormancy regulon expression was assessed by a Green Fluorescence Protein (GFP)-based reporter assay. AA was added to *M. tb* cultures to maintain exogenously added iron in the reduced state (Text S1). Intriguingly, within 4 hr of the addition of 5–10 mM AA to control cultures, *M. tb* exhibited a ∼6-fold induction of the *Rv3134c* promoter (of *Rv3134c-devRS* operon). Both Δ*devR* (Fig. 1) and Δ*devS*Δ*dosT* double mutants (not shown) were unresponsive confirming that the response to AA was mediated through the DevRS/DosT signaling pathway. AA did not modulate the activity of the *sigA* promoter (not shown). *sigA* encodes the principal sigma factor SigA and it is not part of the DevR regulon. Thus AA specifically induces *M. tb* DevR-regulated gene expression rapidly in a concentration-dependent manner. *M. tb* were exposed to AA concentrations that were significantly lower (<10 mM) than those at which chelation-like chemical effects of AA could also play any role (50 mM or higher) [35]. We next assessed the effect of ascorbate derivatives on the DevR regulon. Gene expression was well induced by AA (Figure S1) but not majorly by dehydro- ascorbic acid (DHA, oxidized form of AA) or by magnesium L-ascorbyl 2-phosphate (APM, a slow-releasing ascorbate derivative). Thus reduced AA, at a concentration presumably not attained by addition of APM, is crucial for gene induction.

**Figure 2:**
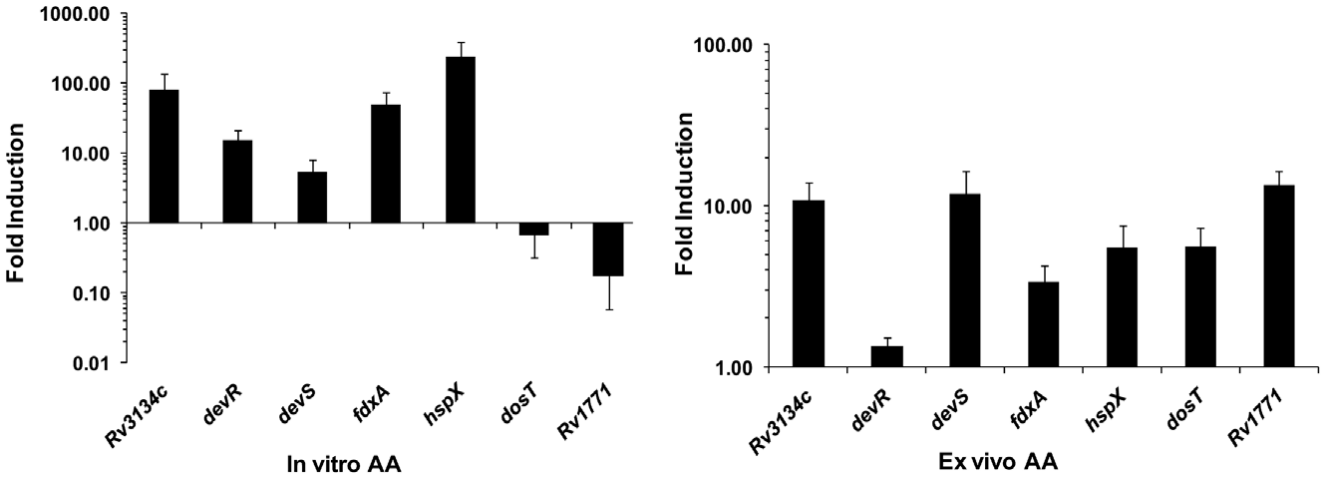
AA-mediated induction of *M. tb* DevR regulon genes. Real time RT-PCR analysis of selected DevR regulon genes was carried out with RNA isolated from *M. tb* H37Rv cultures treated in vitro with AA and *M. tb* RNA isolated from infected THP-1 cells that were treated with AA post-infection (8 hour exposure to AA for both). ‘In vitro AA’, fold induction of individual gene transcription is a ratio of 16S rRNA-normalized transcript number in AA-treated vs. untreated control cultures. Results are expressed as mean ± SD of 3 independent cultures each analyzed in 2-4 replicate assays. ‘Ex vivo AA’, fold induction of individual gene transcription is a ratio of 16S rRNA-normalized transcript number in AA-treated infected THP-1 cells vs. untreated infected THP-1 cells. Results are expressed as mean ± SD of pools of 10 independent infections, each pool analyzed in triplicate.

The discovery of AA-mediated induction of the *Rv3134c* promoter was pursued to determine if exposure to AA could also induce other genes of the dormancy regulon. Real time Reverse transcription (RT)-PCR analysis of RNA demonstrated that in vitro AA treatment induced the expression of all analyzed DevR regulon genes by ∼5- to 240-fold relative to untreated cultures (Fig. 2). No induction was observed in a Δ*devR* mutant, rather, the genes of the DevR regulon were strongly repressed (Figure S2), suggesting that the transcription was DevR dependent. To decipher whether gene expression was also modulated within the intracellular milieu, *M. tb* gene expression was examined in AA-treated vs. untreated infected THP-1 cells by real time RT-PCR. A ∼6- to 13-fold induction of most DevR regulon genes was observed under these ex vivo conditions (Fig. 2). Thus AA rapidly modulates the expression of DevR regulon genes not only in vitro but also within infected cells (ex vivo). The expression of *dosT* was unchanged in AA-treated bacterial cultures but induced over 5-fold in the AA-treated THP-1 cells suggesting a response to an unidentified intracellular stimulus (Fig. 2). The unchanged expression of *dosT* in AA-treated cultures is consistent with the observed lack of *dosT* induction under hypoxia [12]. The underlying similarity between hypoxia and AA treatment is described below.

### O_2_ scavenging by AA underlies DevR regulon induction

**Figure 3:**
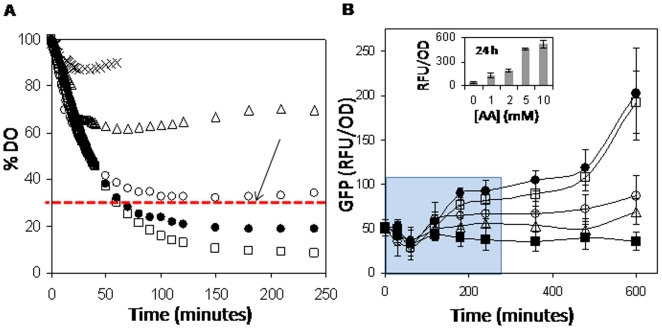
AA-mediated O_2_ scavenging results in induction of DevR-regulated promoter activity. (A) Time dependent depletion of dissolved O_2_ (DO) in Dubos medium with AA concentrations of 1 mM (Δ), 2 mM (○), 5 mM (•), 10 mM (□), 10 mM DHA (×), expressed as% of initial value (saturation). (B) *Rv1738* promoter activity leads to induction of GFP fluorescence (expressed in RFU/OD) in AA-treated shaking *M. tb* cultures. Data is shown as mean ± SD (n = 6) for 0 mM (▪), 1 mM (Δ), 2 mM (○), 5 mM (•), 10 mM (□) of AA. Inset in (B) shows promoter activity in AA-treated cultures at 24 h. The first time point showing a statistically significant induction is shown by the red arrow on the red line in (A). The data that was subjected to statistical analysis is shown in a blue shaded rectangle.

Because AA is reported to have potent O_2_ scavenging activity [36], we reasoned that rapid induction of the DevR regulon could be mediated by the O_2_ depleting function of AA. Accordingly, we monitored AA-mediated depletion of dissolved O_2_ (DO) in Dubos medium. DO is expressed as % DO, where 100% indicates saturation value of the medium and can be assumed to be ∼7 mg/L [37]. DO is depleted in AA- and not DHA-treated medium in a concentration-dependent manner (Fig. 3A). The kinetics of AA-mediated promoter induction was assessed by GFP reporter fluorescence measurements (Fig. 3B). The earliest time point and lowest AA concentration at which the promoter is significantly induced is 180 minutes at 2 mM AA (p<0.05) as determined by two tailed homoscedastic t-tests between fluorescence values at individual time points and the respective baseline (t = 0) values for each AA concentration, which corresponds to a DO concentration of ∼30% (65 µM O_2_, red dotted line, Fig. 3A). A DO concentration of 65 µM is therefore concluded to be the threshold O_2_ concentration at or below which the regulon may be induced. The DO vs. time data is derived from an in vitro experiment (without *M. tb* culture). However, it is reasonable to assume that under shaking conditions cellular metabolic activity likely results in O_2_ depletion to a concentration that is *lower* than the extrapolated value of 65 µM. Therefore we like to state the O_2_ threshold for DevR regulon induction to be <65 µM. Because ∼65 µM threshold DO concentration was attained in ∼100 minutes in the presence of 2 mM AA (Fig. 3A), there is a lag of 80 minutes between attaining the inducing threshold and observing promoter induction. This lag is attributed to the cascade of events comprising the hypoxia response, namely, sensor heme deoxygenation, kinase activation, DevR activation, transcriptional induction and translation. Only negligible GFP expression was observed in the DHA-treated *M. tb* cultures (not shown). Because the effect of AA may have been due to its ability to generate H_2_O_2_ (Text S1), it was tested as a potential inducing signal; DevR regulon induction was not observed in *M. tb* cultures exposed to H_2_O_2_ [10 mM] (not shown) and therefore it was excluded as an inducing signal and the observed regulon induction was ascribed to the O_2_ scavenging by AA.

We also measured the decline in absorbance of methylene blue (MB) which is routinely used for measuring O_2_ depletion [6], [38] to confirm O_2_ depletion by AA. The decrease in MB absorbance as a function of [AA] (Figure S3A) appeared to approximate the directly measured decrease in DO (Fig. 3A). In contrast, DHA reduces MB only mildly (Figure S3B). An inverse dependence of MB absorbance on AA-mediated depletion of DO was noted upon re-plotting the MB absorbance data as a function of the DO data (Figure S3C) and the intercepts in Figure S3D show that MB absorbance works as a reasonably good DO indicator in the presence of AA on at least a qualitative/semi-quantitative level.

### Differential response of DosT and DevS sensors to AA and hypoxia in standing cultures

**Figure 4:**
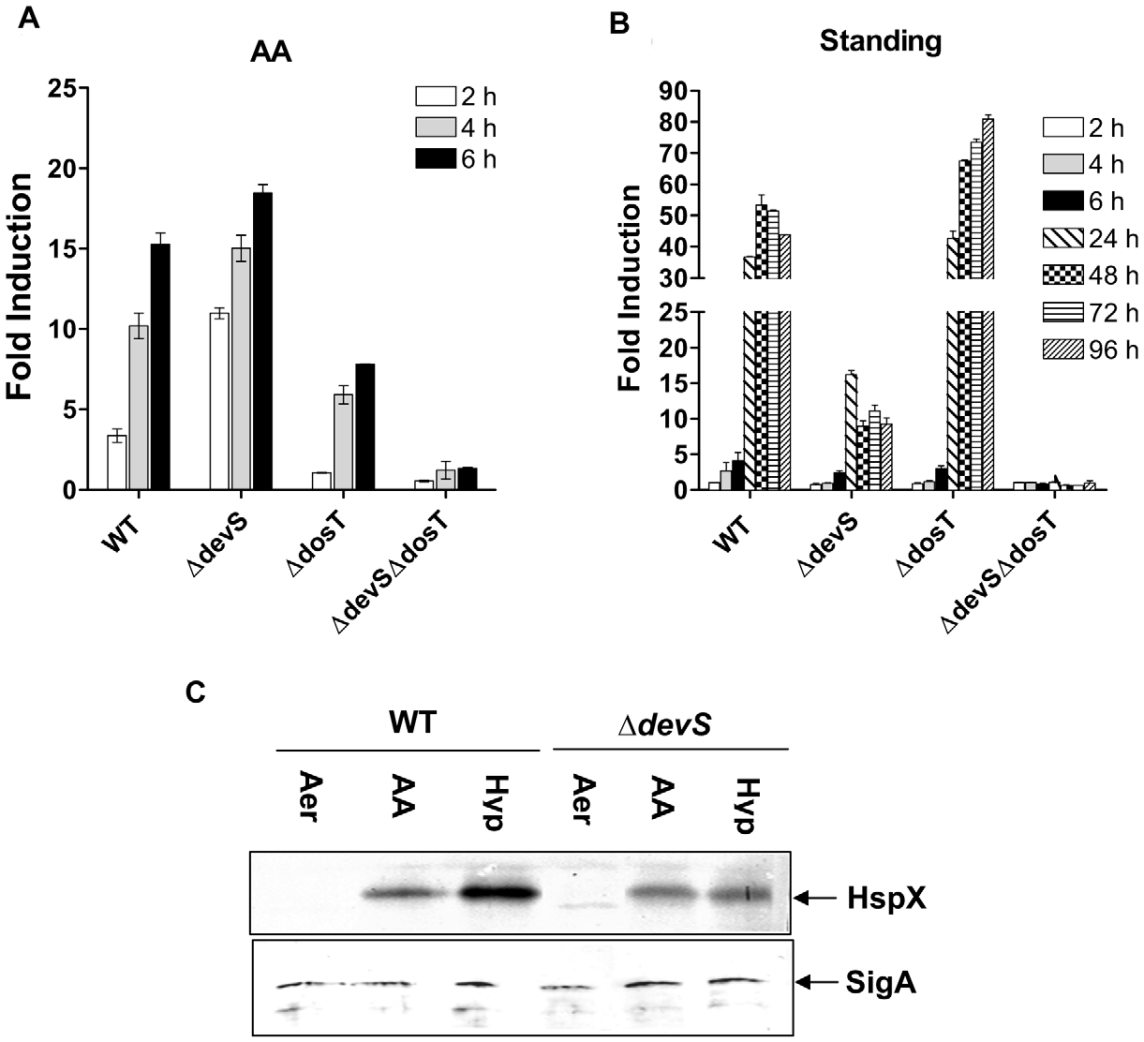
Differential signaling through DevS/DosT by AA and under gradual hypoxia. Induction of *Rv3134c* promoter activity (GFP fluorescence in RFU/OD) was assessed in *M. tb* cultures treated with (A) 10 mM AA, or (B) standing for various intervals of time. Fold induction is the ratio between GFP fluorescence (in RFU/OD) in treated vs. untreated cultures, all in 96-well microplate standing format. (C) Western blot analysis of lysates for expression of HspX and SigA proteins in aerobic *M. tb* cultures (Aer) and those exposed for 8 h to 10 mM AA (under shaking aerobic conditions) or in 5-day standing (Hyp) cultures. Results are expressed as mean ± SD of 3 independent experiments. Note that the y-axis range is different in (A) and (B) panels.

To assess the role of individual kinases in the AA response, *M. tb* mutants lacking *devS*, *dosT* or both sensors (*ΔdevS* Δ*dosT*) harboring *gfp* reporter constructs were used. DosT, and not DevS, supported initial induction of the *Rv3134c* promoter. Cells having DosT (i.e. *devS* mutant) showed ∼10-fold induction within 2 h of exposure to AA, whereas those with DevS (i.e. *dosT* mutant) failed to support promoter induction in the presence of AA during the same period (Fig. 4A). The differential response of the mutants was noted over the entire concentration range of AA that was tested (not shown).

Because the DevRS/DosT pathway is hypoxia responsive and induces the DevR dormancy regulon [7], we compared the ‘AA’ and standing hypoxia culture models for the kinetics of DevR regulon induction and the role of individual sensors (Fig. 4A and 4B). Under standing conditions, promoter activity was strongly induced only at 24 h and beyond due to the gradual development of hypoxia. Induction occurred with differential kinetics and to varying extents; ∼18-fold and 7-fold in AA-treated vs. poor induction in standing cultures of Δ*devS* and Δ*dosT* mutants at 6 h. Upon further standing (24 h and 96 h), cells expressing DevS showed a robust induction response of ∼43- to 82- fold in contrast to cells expressing DosT which showed a weaker and diminishing response of ∼15- to 9- fold. The kinetics of regulon induction in the mutant strains suggests that rapid hypoxia development by AA enables an initial DosT response and gradual O_2_ depletion (in standing culture) majorly supports signaling via the DevS sensor, respectively. The induction of the dormancy regulon was confirmed at the translational level; HspX was induced under all treatment conditions (Fig. 4C) and SigA level was maintained constant under all conditions consistent with a previous report [39].

### Ascorbic acid also induces the expression of acid-responsive and antioxidant genes in *M. tb*

**Figure 5:**
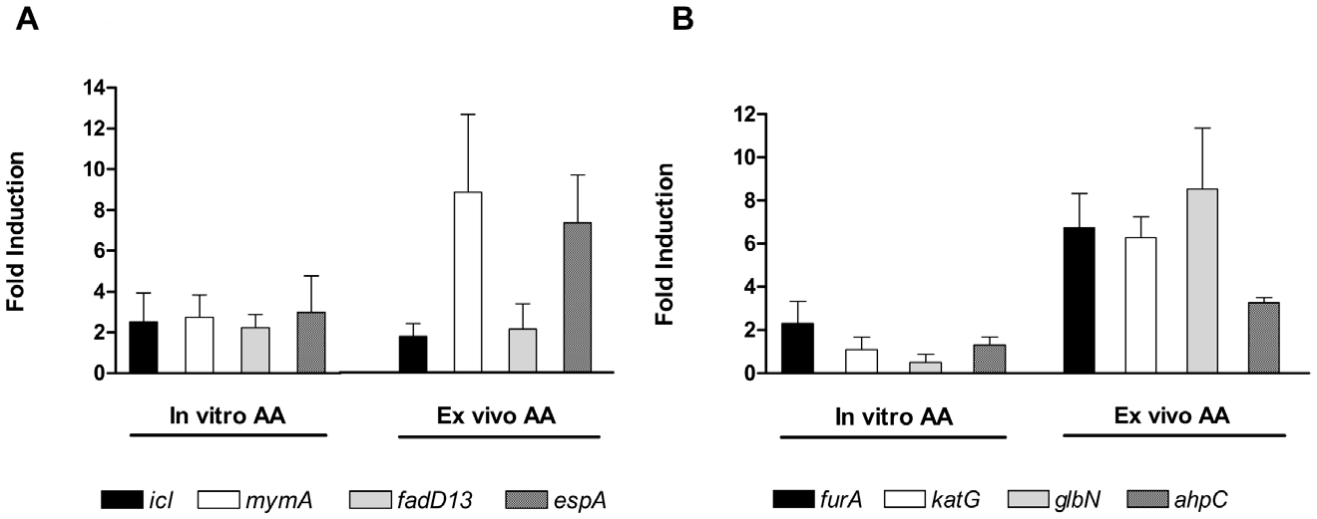
AA-mediated induction of *M. tb* acid response and antioxidant response genes. Experimental details are described in Fig. 2legend and Experimental procedures.

Because the addition of AA causes the acidification of Dubos medium to pH ∼5.5, we asked whether acidification per se modulated the dormancy regulon response. The exposure of *M. tb* to pH 5.5 in Dubos medium (buffered with acetic acid) did not induce the dormancy regulon, thereby ruling out a low pH response for this regulon (not shown). We also asked whether the AA-mediated induction of the DevR regulon is pH dependent or not. When AA was buffered and added to Dubos medium (final pH of the medium was 6.5), the DevR regulon promoter was still induced in a similar manner (not shown). We did find, based on real-time RT-PCR analyses of a few target genes selected on the basis of published reports of their acid-inducibility that AA caused some of them to be induced. For example, *icl* (encoding isocitrate lyase, a key enzyme of the glyoxylate pathway), *mymA* and *fadD13* genes of the *mymA* operon (*Rv3083–3089*), that plays a role in mycolic acid biosynthesis [40], [41] and *espA*/*Rv3616c* of the *Rv3616c–Rv3614c* operon that encodes the ESX-1 protein secretion system [42] were induced >2-fold in AA treated *M. tb* cultures (Fig. 5A). Their expression was enhanced ∼2- to 9-fold within THP-1 cells post exposure to AA, relative to untreated infected THP-1 cells, which suggests that the intracellular pH is acidic in AA-treated cells. The pH response was independent of DevR with the exception of *fdxA* which is a member of the DevR regulon (Figure S2) and whose expression is acid inducible in *M. tb* H37Rv as reported earlier [40].

Next, because AA is thought to form H_2_O_2_ in the presence of molecular oxygen, we also tested whether AA induced the expression of selected antioxidant genes reported to be H_2_O_2_ responsive. *ahpC* and *katG* are among the best studied mycobacterial genes that afford protection against reactive oxygen and nitrogen intermediates; *katG* encodes catalase and is genetically linked with *furA* that encodes an iron-regulated repressor and the *furA-katG* operon is reported to be induced by H_2_O_2_ [43], [44]. All the genes analyzed namely, *furA*, *katG*, *glbN* and *ahpC*, showed an AA response and were induced ∼3- to 8-fold within THP-1 cells compared to untreated infected cells (Fig. 5B) and the in vitro transcriptional response was largely independent of DevR (Figure S2). There was mostly no induction of antioxidant genes after addition of AA in vitro. Note that the *M. tb* transcriptional response to AA was assessed at a single time point (8 hr) and may not reflect the entire dimension of the bacterial response. The induction in gene expression (Fig. 2 and Fig. 5) observed in AA-treated THP-1 cells is with respect to untreated THP-1 cells infected with *M. tb* and establishes AA as a likely intracellular signal.

### AA treatment triggers bacteriostasis and bacterial dormancy phenotype development

**Figure 6:**
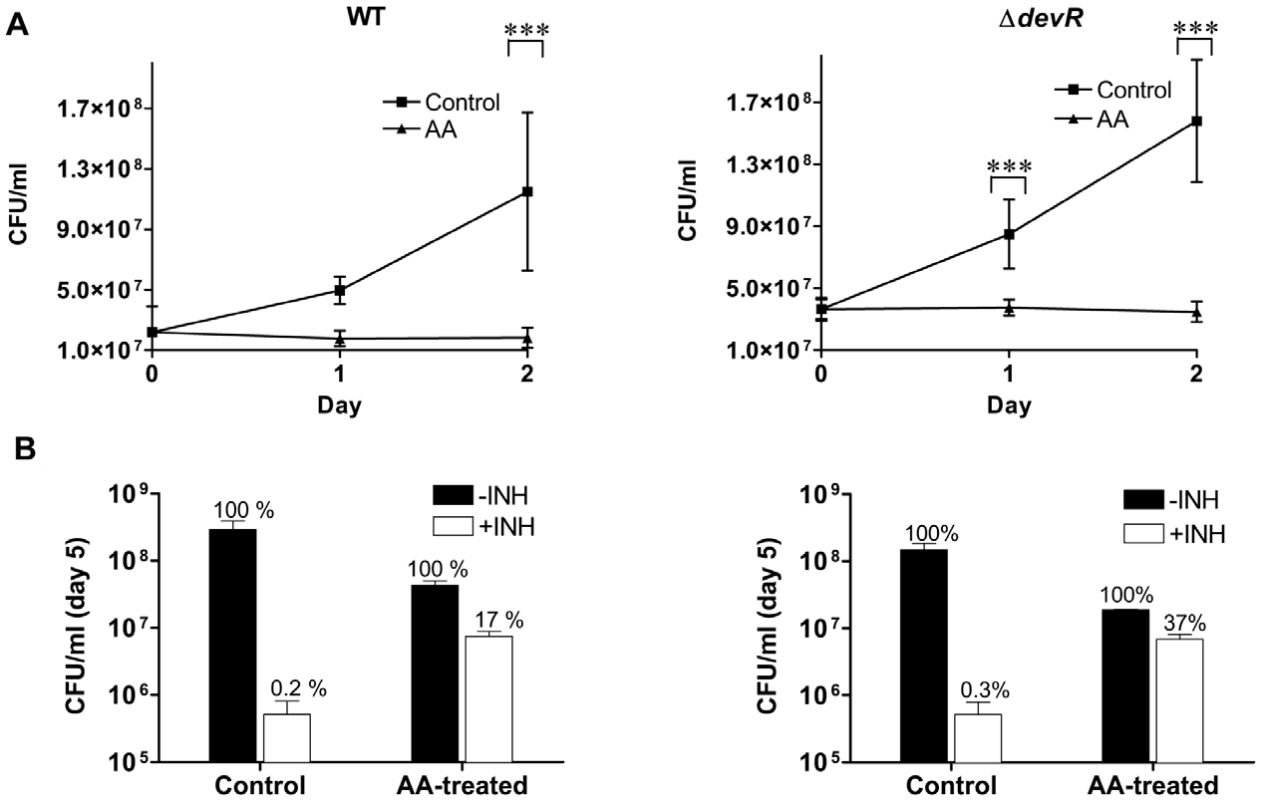
AA-triggered bacterial growth arrest and dormant physiology in axenic cultures of *M. tb*. (A) Bacteriostasis of AA-treated aerobic *M. tb* cultures. Statistically significant difference in CFUs recovered from AA-treated vs. untreated (Control) aerobic cultures was determined using paired two-way ANOVA with Bonferroni post-test correction (***, p-value <0.001). (B) INH tolerance of AA-treated aerobic *M. tb* cultures. Cultures were exposed to 10 mM AA for 1 day followed by treatment with INH (4 µg/ml) for 4 days. The number of viable bacteria on day 5 is shown. CFU in ‘-INH’ ‘Control’ (untreated) or ‘AA-treated’ wells is depicted as 100%; bacteria surviving INH treatment are depicted as ‘+INH’ with respect to their controls (100%). Mean ± SD of 3 independent cultures is shown.

Because AA induces the expression of the DevR regulon by generating hypoxia, a condition known to trigger ‘dormancy’ in *M. tb* [6], we next asked whether AA affected the growth, viability and physiology of *M. tb* cultures. At 10 mM concentration, which approximated the intracellular concentration attained within activated macrophages [31], [32], [45], bacteriostasis was induced in AA-treated aerobic cultures of *M. tb* WT and sensor kinase mutant strains; while untreated cultures of all the strains grew up to ∼3- to 6-fold under similar culture conditions over a 2 day period (Fig. 6A and Figure S4A). Because DevR regulon induction occurred early and preceded the cessation of mycobacterial replication, AA could have potentiated the adaptation of actively growing bacteria to a stage of growth arrest, similar to that observed in other hypoxia-based models of dormancy. Untreated *M. tb* strains grew ∼5- to 16-fold over 5 days and were INH susceptible. By contrast, AA-treated cultures were growth arrested and a significant proportion of AA-treated cultures were tolerant to INH at a concentration that was 10 fold higher (4 µg/ml) than the MBC (0.4 µg/ml) of INH for *M. tb* H37Rv (17%, Fig. 6B and Figure S4B). INH is known to specifically kill multiplying bacteria and is not bactericidal against non-replicating dormant bacilli [4], [21], [46], while RIF kills both replicating and non-replicating mycobacteria. No bacilli were recovered from RIF-treated cultures (data not shown). Therefore, a significant fraction of AA-treated in vitro bacteria acquire phenotypic resistance to INH together with a growth arrested phenotype. Remarkably, abrogation of DevRS/DosT signaling in mutant strains failed to abolish the INH resistance phenotype of AA-treated cultures; ∼37–39% of bacteria were viable and INH tolerant (Figure S4). The viability of AA-treated bacteria was also confirmed by green staining with fluorescein diacetate (not shown).

Because low pH is known to cause *M. tb* growth arrest and AA acidifies the medium (Dubos medium has a pH of ∼5.5 in the presence of 10 mM AA), *M. tb* viability was assessed in the presence of AA that was buffered to provide a pH of ∼6.5 in Dubos medium in order to determine if the AA effect was pH dependent. The growth arrest phenotype was also noted in the presence of buffered AA thereby establishing the growth arresting property of AA to be pH independent (data not shown). Because exposure to DETA/NO leads to DevR regulon induction, we also examined its effect on bacterial growth. In contrast to the bacterial response to AA, treatment of *M. tb* cultures with DETA/NO failed to arrest bacterial replication or confer phenotypic resistance to INH (Figure S5). Our findings are consistent with earlier reports that DETA/NO treatment at low concentrations did not have a bacteriostatic or bactericidal effect [5].

### Ascorbic acid impairs *M. tb* growth and induces development of dormancy phenotype in THP-1 infection model

**Figure 7:**
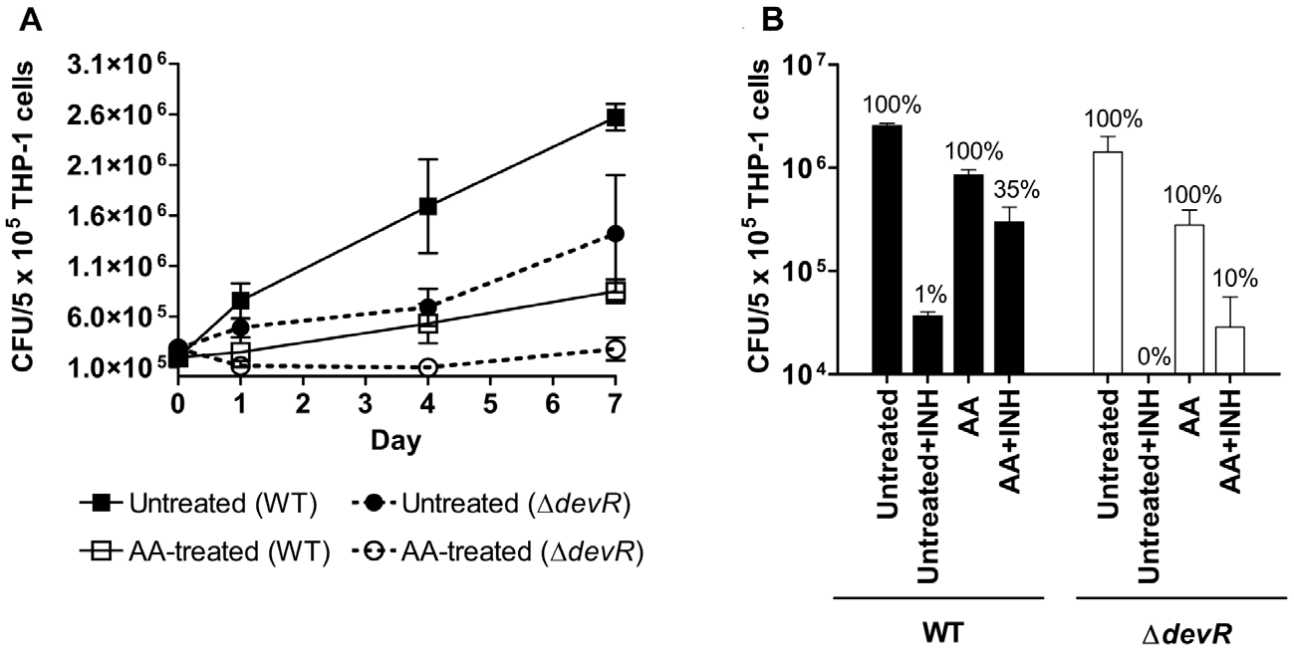
AA-triggered bacterial growth arrest and dormant physiology of *M. tb* in infected THP-1 cells. (A) Bacteriostasis within untreated and AA-treated THP-1 cells. In case of ‘AA’-treated THP-1, the cells were treated with AA immediately after bacterial infection (i.e. day 0). Statistically significant difference in CFUs recovered from AA-treated vs. untreated THP-1 cells on various days was determined using paired two-way ANOVA with Bonferroni post-test correction (***, p-value <0.001). (B) Dormant physiology of *M. tb* within AA-treated THP-1 cells. Infected THP-1 cells were either not treated (‘Untreated’) or treated with 2 mM AA (‘AA’) for 7 days post-infection. INH (4 µg/ml final concentration) was added on day 1 to ‘Untreated+INH’ or ‘AA+INH’cultures, and INH treatment continued until day 7. Viable counts were determined on day 7 post-infection (i.e. 6 days post INH-treatment). ‘Untreated’, 100%, total bacteria on day 7; ‘Untreated+INH”, % bacterial recovery with reference to ‘Untreated’; AA, 100%, total bacteria on day 7; ‘AA+INH’, % bacterial recovery with reference to ‘AA’. Mean ± SD of 3 independent experiments is shown.

Based on analysis of gene expression, growth arrest and tolerance to INH, we show that in vitro vitamin C treatment triggers a phenotype, called dormancy phenotype, which might mimic the state of the tubercle bacillus during latent TB. To assess whether the dormancy phenotype was also exhibited by intracellular *M. tb*, infected THP-1 cells were treated with AA post-infection and its effect on bacterial growth and INH phenotype was assessed. Control *M. tb* WT bacteria grew 9-fold over a 7-day period in contrast to AA-treated cultures that were growth arrested (Fig. 7A). INH treatment of control WT bacteria led to a ∼99% reduction in their intracellular viability on day 7 indicating that they were majorly in the replicating phase and therefore susceptible to the bactericidal action of INH (Fig. 7A and B). Though intracellular growth of the *devR* mutant was relatively sluggish over the same period (approximately 5-fold), these bacteria were also noted to be INH sensitive (Fig. 7B). However, both WT and Δ*devR* bacteria exhibited an INH tolerant phenotype when they were exposed to AA within THP-1 cells (∼40% and 57%, respectively on day 4, not shown). Fewer AA-treated Δ*devR* bacteria sustained the INH tolerant phenotype on day 7 (10% of Δ*devR* bacilli vs. ∼35% of WT, Fig. 7B). However because no INH tolerant mutant bacteria were recovered from untreated cultures, we cannot conclude on whether AA is more effective at producing INH-tolerant phenotype in WT bacteria than mutant organisms. Intracellular AA-treated bacilli were confirmed to be viable by fluorescein diacetate staining (not shown).

## Discussion

The DevR/DevS/DosT system has been the subject of intense investigation and is the most widely studied two-component signal transduction pathway in *M. tb*. The serendipitous discovery of vitamin C as an inducer of the *M. tb* DevR regulon is novel as it implicates an essential nutrient as a possible stimulus for the first time. We further elucidated that regulon activation was a consequence of rapid hypoxia development due to the O_2_ scavenging properties of vitamin C. Moreover, vitamin C exposure appears to mimic at least some of components of the intracellular scenario wherein *M. tb* is exposed to multiple stresses (hypoxic, oxidative, nitrosative, acidic etc.). The broad modulatory effects on gene expression are accompanied by bacterial growth arrest and development of the dormancy phenotype. These adaptations occur not only in in vitro cultures but also in THP-1 cells and establish a vitamin for the first time as a trigger of bacterial growth arrest and dormancy phenotype development in *M. tb*.

**Figure 8:**
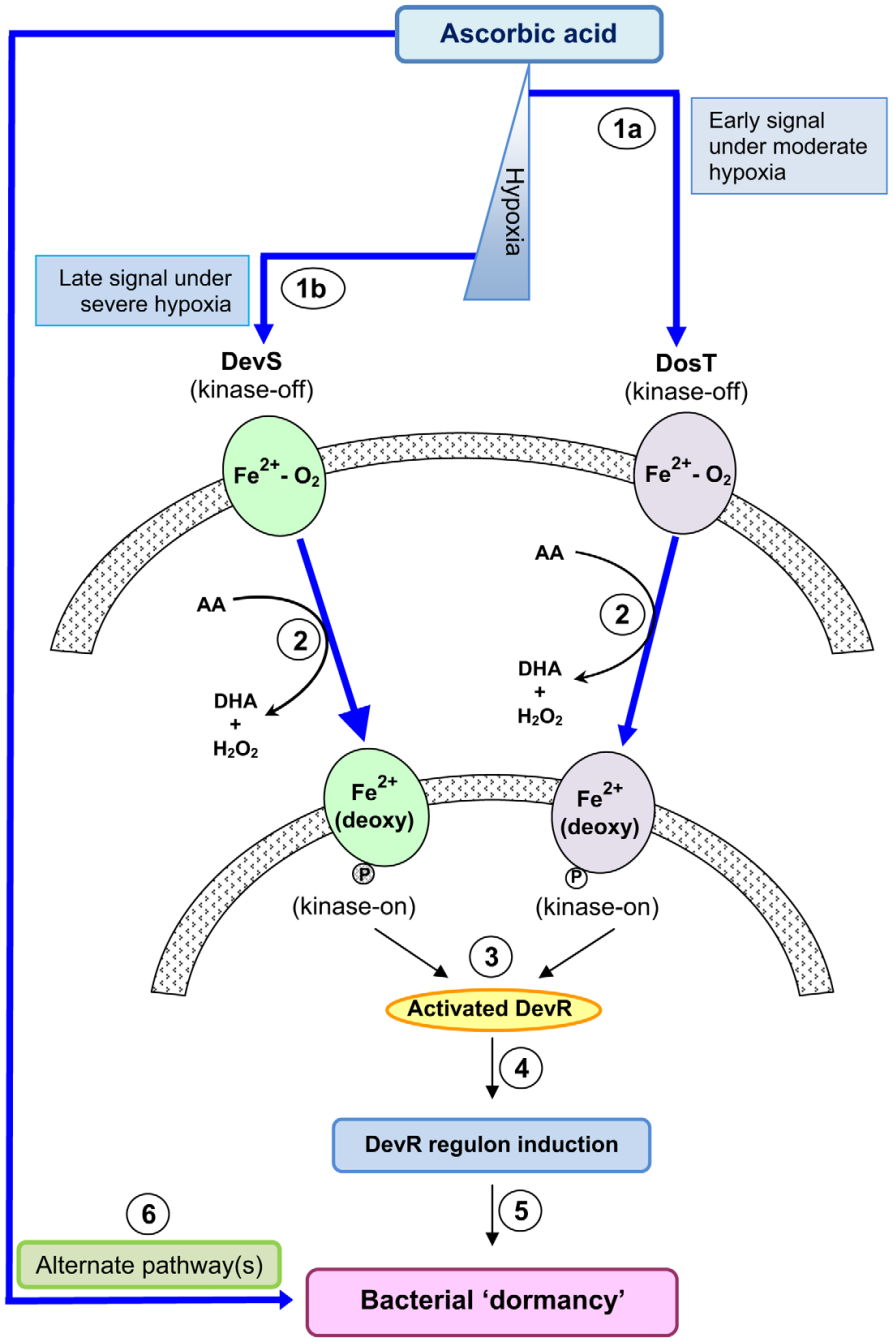
*M. tb* ‘dormancy’ response to ascorbic acid. In one pathway, hypoxia generation by AA is sensed by DosT and DevS resulting in their activation (first deoxy-DosT and then deoxy-DevS under progressive hypoxia, steps 1a, 1b and 2). The transfer of phosphosignal from the sensors to DevR results in its activation (3). Activated DevR binds to target gene promoters and triggers DevR regulon mechanisms (4) and bacterial ‘dormancy’ (5). Bacterial ‘dormancy’ is also attained through alternate pathway(s) upon exposure to vitamin C (6).

The need for two kinases, namely DevS and DosT, to activate DevR has been raised by us and others [10]–[12], [16]. Some suggestions that were made to explain the presence of two sensors were that they could serve a redundant function, or they could belong to different physiological states or they may enable a pathogen response to various O_2_ concentrations or cations which could be achieved through two sensors of different properties. Genetic analysis of individual sensor kinase mutants provided important insights into the relative roles of DevS and DosT during *M. tb* adaptation in the ‘AA’ and hypoxia models. Our observations can be rationalized on the basis of the O_2_ binding properties for purified DevS and DosT proteins that were described by several groups. While DosT is consistently characterized as an O_2_ sensor [14], [16], DevS was identified as a gas sensor by some groups [13], [16], [47], [48] and proposed to be a redox sensor by others although this was not based on measurement of its redox potential [14], [49]. Moreover, DevS was very stable to autoxidation and not likely to exist in the met (Fe^3+^) state in vivo [49], a condition that is necessary for a redox sensing function. As DosT binds ∼8-fold less tightly to O_2_ than DevS [16], a progressive decline in DO concentration is predicted to elicit an initial DosT response by switching its kinase activity first when deoxygenated to the Fe^2+^-deoxy form, followed by a DevS hypoxic response. The GFP reporter assays indeed revealed an initial DosT response followed by a robust DevS-mediated hypoxic response that exceeds the DosT response. The initial response of DosT, and not DevS, to vitamin C establishes that DosT is phosphorylated first in vivo. A similar response, first by DosT and then DevS, was also reported recently in a standing hypoxia model [50]. Thus the observed differential response of DosT and DevS kinases to O_2_ provides a plausible justification for the involvement of two sensors with distinct properties during *M. tb* adaptation to progressive hypoxia (Fig. 8).

AA consistently elicited a strong initial DosT response in the Δ*devS* mutant vs. the WT strain (Fig. 4A). This suggests that DevS dampens the DosT response in an unknown manner. Despite this effect, initial DosT- mediated signaling is efficient [likely reasons are that DosT is activated (deoxygenated) first, and DosT has a higher initial rate of phosphorylation, [13], [16]. The powerful regulon response in the Δ*dosT* mutant (but not in Δ*devS* mutant) from 24 hours onwards (Fig. 4B) establishes that the initial DosT response is sufficient to elicit an amplified DevS response that is essential in the face of progressive hypoxia development. On the basis of our findings, the powerful and sustained regulon response in *M. tb* is attributed to DevS via positive autoregulation of *Rv3134c-devRS*, and not to DosT, whose expression is not induced during hypoxia [12], [51].

We correlated the differential response of the kinases to O_2_ with the O_2_ saturation limits reported for NRP-1 and NRP-2 stages of bacterial adaptation in the Wayne dormancy model [46]. The threshold O_2_ concentration for inducing the DevR regulon (∼30%) was higher than that associated with bacterial transition into NRP-1 at ∼1% O_2_ saturation [46]. DevS activation is also expected to occur at a higher O_2_ concentration at∼3 µM [16] than that at which bacteria enter into NRP-2 at∼0.06% saturation [46]. On this basis, we conclude that *both* kinases are switched on *prior* to and not *during* NRP-1 and NRP-2 stages and therefore we propose that the DevR regulon is activated early on under moderate hypoxia to prepare the bacteria for adaptation and survival under more severe hypoxia encountered later during NRP-1 and NRP-2 stages. Hypoxia clearly results in growth arrest and AA is unique among dormancy regulon-inducing signals as it triggers rapid growth arrest and confers a dormancy phenotype (phenotypic tolerance to INH) unlike NO and CO. The ‘AA’ model differs from the Wayne model in the rate at which hypoxia is established; AA scavenges O_2_ rapidly to create hypoxia within few hours, while O_2_ is depleted gradually over a few days as a consequence of *M. tb* respiration in the Wayne model [46]. In addition to scavenging O_2_, AA is also expected to bring about a rapid change in the redox potential. In this context it is noteworthy that a decline in the redox balance (NAD^+^/NADH ratio) of *M. tb* cultures was observed during hypoxia development [52]. A redox response is not specific to any single reaction but rather closely coupled with a variety of steps in cellular metabolism. Leistikow and colleagues suggested a role for DevR regulon mechanisms in maintaining redox balance in hypoxic *M. tb* cultures and further noted that induction of the regulon precedes inhibition of respiration and may facilitate bacterial preadaptation to the forthcoming nonrespiring state [52]. Additionally, the hypoxic threshold for regulon induction was attained with 2 mM AA which is expected to exert a slower effect on redox change compared to 10 mM AA (see methylene blue data, Figure S3 panel A), further indicating the role of O_2_ depletion, rather than other redox effects, in inducing the regulon. Therefore, although *M. tb* exhibits a redox response to hypoxia, it *follows* the regulon response, is unrelated to the sensing function of DevS or DosT, and appears to be a downstream effect typical of slowed metabolism in cells.

A significant feature of the vitamin C-mediated response is the growth arrest and dormant phenotype development of both WT and *ΔdevR* mutant bacteria. Our findings suggest that this phenotype must be due to (an) other unidentified effect/s of vitamin C treatment on *M. tb* gene expression and/or physiology. The induction of some acid response genes suggests that *M. tb* are exposed to a low pH within AA treated THP-1 cells and therefore a pH stress could also have triggered intracellular growth arrest as reported earlier [1], [53]. Future investigations are necessary to obtain further insight into the mechanism(s) underlying growth inhibitory effects and ‘dormant’ phenotypes induced in *M. tb* by AA treatment. Due to its various properties, vitamin C is likely to exert pleiotropic effects thereby influencing host gene expression/signaling pathways and these effects could influence the outcome in a DevR independent fashion. For example, vitamin C downmodulates signaling of GM-CSF, a pro inflammatory cytokine, and also inhibits NFkB activation by diverse stimuli [54], [55], suggesting a role for it in anti-inflammatory responses and in immune defense.

Much of our current understanding of *M. tb* infection is derived from ex vivo infection models wherein *M. tb* undergoes multiplication [56]. However, AA deficiency is likely to occur in cultured cells due to its rapid depletion in commercial media [33]. Although the levels of AA in human serum, plasma and immune cells range from 0.1 mM–0.2 mM [32], activated monocytes/macrophages and neutrophils are known to concentrate AA to much higher levels that equal or even exceed 14 mM [31]–[34]. On the basis of the observed responses, namely, bacterial growth arrest, phenotypic dormancy phenotype (INH tolerance) and modulation of gene expression in AA treated THP-1 infected cells (vs. untreated THP-1 infected cells), we believe that *M. tb* is likely to be exposed to modulatory concentrations of AA in the THP-1 cell infection model.

A striking connection between vitamin C and TB is that both humans and guinea pigs are highly susceptible to TB and require vitamin C supplement in the diet, as they lack L-gulono-1,4-lactone dehydrogenase/oxidase (Gulox/Rv1771), the terminal enzyme in the AA biosynthetic pathway. Intriguingly, *M. tb* produces this enzyme and has the capacity to synthesize AA [57]. The expression of *Rv1771* transcripts was repressed in AA-treated *M. tb* cultures suggesting the possibility of feedback inhibition of this cascade in vitro. However, gene induction resulting from exposure to AA under ex vivo conditions is consistent with differential regulation in intracellular vs. in vitro cultured bacteria (Fig. 2 and Figure S2).

The novelty of the present findings rests in the discovery that vitamin C has wide-ranging regulatory effects on *M. tb*. Ascorbic acid modulates not only DevRS/DosT signaling and elicits a low pH and oxidative stress response but also arrests bacterial growth. These effects occur in axenic culture as well as under ex vivo conditions. In addition to these effects on bacterial transcription and growth, AA also renders the bacteria INH tolerant, a phenotype associated with dormant organisms. Therefore, at a practical level the ‘AA dormancy-infection model’ offers a potential alternative to other models of non-replicating persistence of *M. tb* and may find application for investigating host-‘dormant’ *M. tb* interactions without the inherent limitations of axenic cultures (such as the hypoxia and nutrient starvation models) or the complexities of an animal model. Note that except for guinea pigs, other animal models are not useful as they synthesize AA.

To the best of our knowledge there is no description in the literature of an essential dietary compound which elicits genetic and physiological responses in *M. tb* as broad in scope as vitamin C. Our findings suggest the possibility of anti-bacterial activity of vitamin C, a relatively neglected chapter in the impact of nutritional factors on TB. It is plausible that the positive association between vitamin C and recovery from TB noted in the pre-antibiotic era perhaps could be attributed, at least in part, to the vitamin's bacterial growth arresting and/dormancy promoting properties. In the current scenario of TB disease, AA could (a) modulate the efficacy of antitubercular regimens (that target replicating organisms) and, (b) contribute to TB control among immunocompromised individuals such as HIV subjects. Our findings provide a new and testable hypothesis to decipher the possible protective role of this vitamin in TB that extends beyond the popular link between vitamin C and host immunity. These possibilities will undoubtedly require rigorous testing in animal model and human subjects.

## Materials and Methods

### Bacterial strains, plasmids and culture

*M. tb* H37Rv was cultured in Dubos medium containing 0.5% BSA, 0.75% Dextrose and 0.085% NaCl plus 0.15% Tween-80 (DTA medium) with shaking at 220 rpm at 37°C. *M. tb* mutant strains Δ*dosR*/Δ*devR*, Δ*dosS*/Δ*devS,* Δ*dosT*, Δ*dosS*Δ*dosT*/Δ*devS*Δ*dosT* [11] were a generous gift from Dr. David Sherman (Seattle Biomedical Research Institute, USA). These strains were electroporated with a DevR-regulated promoter-based GFP reporter plasmid (Table S1). Hygromycin was used at 50 µg/ml concentration when required.

All strains were expanded from −70°C frozen stocks before the experiment and subcultured twice in DTA medium as described above. GFP reporter assays were performed by diluting twice sub-cultured bacteria (OD_595_ ∼0.3) to OD_595_ ∼ 0.1 in DTA medium and dispensing 200 µl culture aliquots in a 96-well microplate. AA was added to the wells as required and the plates were incubated under standing conditions at 37°C. Promoter activity was assessed by measuring GFP fluorescence at different time points as described [51] after subtracting background fluorescence from AA and empty vector control plasmid wells. To assess GFP reporter activity under shaking conditions (DO experiments), *M. tb* cultures were diluted to OD_595_ ∼0.1 in Dubos medium (without ADC) and 4 mL aliquots were dispensed in 50 mL tubes in duplicate. AA was added at various concentrations and the tubes were incubated under shaking conditions as described. At each time point, triplet culture aliquots were withdrawn from dedicated tubes in duplicate and GFP fluorescence was measured which is expressed as RFU/OD as described [51].

### Western blotting

Cultures grown as above were treated with 10 mM AA or kept standing for 5 days. Cell lysates were prepared as described [58] and analyzed by immunoblotting using rabbit anti-HspX/anti-SigA polyclonal sera as described [23]. Anti-SigA antibody was a generous gift from Dr. T.S. Balganesh (AstraZeneca, Bangalore).

### RNA extraction and Real time Reverse transcription-PCR

RNA was isolated from *M. tb* cultures that were treated with AA (10 mM) for 8 hours or untreated under shaking conditions at 37°C as described [51]. RNA (500 ng) was reverse transcribed to cDNA and further amplified using gene-specific primers (Table S2) to generate SYBR green-labeled PCR products using MyiQ thermal cycler (Bio-Rad). Reaction conditions were 94°C (10 min) followed by 45 cycles of 94°C (30 s), 65°C (45 s) and 72°C (30 s). Reactions without reverse transcriptase were set up alongside to account for amplification of contaminating DNA if any. Transcript levels between various RNA samples were normalized using 16S rRNA.

### *In vitro* viability assay

*M. tb* strains were grown as described above and aliquoted into two 3 mL portions in 15 mL tubes. AA treated (10 mM final concentration) and untreated cultures were incubated under shaking conditions as described above and aliquots were removed at specified time points for CFU analysis. At 24 h post-AA addition, control and AA-treated cultures were further divided into 3 aliquots; INH (4 µg/ml) and RIF (2 µg/ml) were each added to an aliquot. The tubes' contents were dispensed as 200 µl aliquots in a 96-well microplate, incubated upto 5 days and the contents plated on Middlebrook 7H11 containing 10% ADC (0.5 g/l BSA, 2g/l Dextrose and 0.085 g/l NaCl) (MB) agar for CFU analysis. To assess if the growth arrest property of AA was due to its acidifying action, *M. tb* WT culture was grown in DTA medium as described above, adjusted to an OD_595_ of ∼0.1 and aliquoted in three 3 mL portions into 15 mL tubes - Tube 1, untreated culture control (Dubos medium has a pH of ∼6.5); Tube 2, 10 mM AA-treated culture (final pH of ∼5.5); Tube 3, 10 mM AA-treated cultures (previously buffered to yield final pH of 6.5). The tubes were incubated under shaking conditions and then aliquots were removed at specified time points (day 0, 1 and 2) for CFU analysis.

### THP-1 infection model

THP-1 cells were grown in RPMI 1640 medium supplemented with 10% fetal calf serum and 4 mM L-glutamine. They were seeded in multiple wells of a 24-well tissue culture plate (0.75×10^6^ cells per well) and differentiated by 24 h treatment with 40 nM phorbol 12-myristate acetate. Infections were carried out for 3 h with untreated *M. tb* WT and Δ*devR* bacteria at a multiplicity of infection of 1; the contents of the wells were subsequently washed with incomplete RPMI 1640 medium and incubated in fresh complete RPMI 1640 medium. AA was added to the test wells post-infection (final concentration 2 mM). After incubation at 37°C under 5% CO_2_ for 1 day, INH (4 µg/ml final concentration) was added to a set of untreated and AA-treated wells. The plates were further incubated for 4 and 7 days post-infection. After 4 days of infection, the medium was replenished, fresh AA and/INH were added to the relevant wells and all the infected cultures were incubated further for three days until day 7. Total viable bacterial load was assessed on day 0, 1, 4 and 7 post-infection by lysis of the monolayers in 0.025% SDS, followed by plating dilutions on MB agar. Statistically significant differences in CFUs recovered from AA-treated vs. untreated cultures (in vitro and ex vivo) were determined using paired two-way ANOVA with Bonferroni post-test correction.

### Isolation of *M. tb* RNA from infected THP-1 cells

Twenty 175-cm^2^ flasks were each seeded with 10×10^6^ THP-1 cells that were grown as described above. After 2 days, ∼40×10^6^ cells were differentiated with PMA as described above, infected with *M. tb* H37Rv for 3–4 h at a multiplicity of infection of 10∶1 per flask; washed with incomplete RPMI 1640 medium and incubated in fresh complete RPMI 1640 medium. Thereafter, AA (final concentration of 2 mM) was added to ten flasks (set B). The remaining ten flasks represented the untreated infected culture control (set A). Following an 8 hr treatment with AA, the cells from both the sets were individually scraped and lysed with TRI reagent to release intracellular bacteria. RNA was isolated from the pooled intracellular bacterial pellets (from set A and set B flasks) and subjected to Real time RT-PCR analysis and 16S rRNA normalization as described above. Fold change in transcript levels in AA-treated intracellular bacteria was calculated with respect to those in untreated intracellular bacteria to determine the specific effect of AA on intracellular gene expression.

### Dissolved oxygen measurements

Direct and indirect measurements of DO post-addition of AA were performed using a DO meter (CyberScan DO300 Waterproof DO Meter, Eutech Instruments, Singapore) with a portable probe and methylene blue (MB) redox dye, respectively. The direct DO experiment was performed to determine whether hypoxia develops in the presence of AA and our findings demonstrate that AA deplete O_2_ from the medium in a concentration dependent manner. DO measurements were made in bacterial growth medium (and not in *M. tb* cultures) because we do not have access to probes for monitoring O_2_ levels under biosafety conditions. Briefly, 50 mL Dubos medium (without culture) was stirred in a 50 mL volume beaker at constant temperature (28±1°C) on a magnetic stirrer. The DO meter was calibrated by setting the output reading to 100% value, which was obtained by purging Dubos medium with air, under mild stirring, for ∼10 minutes to attain the saturation limit for DO. Different concentrations of AA (1–10 mM) were added to constantly stirring medium and DO levels were measured at regular short tim intervals up to 4 hours. When used, MB was added to Dubos medium at a final concentration of 3 µg/ml and aliquots were removed from control and AA treated samples incubated under shaking conditions. Decolorization of MB was monitored by the measurement of A_655_ up to 4 hours.

## Supporting Information

Text S1 Some reactions of Ascorbic acid.
(0.07 MB DOC)

Table S1 Plasmids used in this study.
(0.03 MB DOC)

Table S2 Primers used for quantitative reverse transcriptase-PCR analysis.
(0.04 MB PDF)

Figure S1 Reduced AA is crucial for inducing the DevR dormancy regulon. Effect of various AA derivatives, AA, DHA and APM, on gene induction at 6 h is shown. Fold induction is a ratio of Rv3134c promoter activity in AA-treated cultures vs. untreated cultures (standing plate format) and is expressed as mean ± SD (n = 3 independent experiments).
(0.08 MB DOC)

Figure S2 Real time RT-PCR analysis of AA-mediated induction of DevR regulon, acid response and oxidative response genes in M. tb ΔdevR cultures in vitro. Experimental details are described in Fig. 2 legend and Experimental procedures.
(0.10 MB DOC)

Figure S3 Methylene blue (MB) absorbance as a function of dissolved oxygen. (A) MB absorbance (A655) in culture medium under shaking conditions (without cells) decreases with time for AA concentrations of 0 (▪), 1 mM (Δ), 2 mM (○), 5 mM (•), 10 mM (□). (B) DHA does not lead to a substantial decolorization of MB (concentrations of DHA used and symbols are same as those in (a)). (C) A655 plotted as a function of DO can be approximated as a linear relationship (regression coefficients of 0.51, 0.63, 0.82 resp.) of the form A655 = (1.63×10−3±2.52×10−4)DO + Intercept (see below), where DO is expressed in %DO, for AA concentrations of 2 mM (○), 5 mM (•), 10 mM (□). (D) The intercepts from (c) show a linear inverse dependence on [AA] of the form Intercept  = −0.0239[AA] + 0.3503, where [AA] is in mM. The calibration of MB absorbance as a function of dissolved O2 can be simplistically approximated as A655∼0.0016(DO) + (−0.0239[AA] + 0.3503), where DO is expressed as %DO and [AA] is in mM. While the linear relationship is certainly a simplified approximation, at least on a semi-quantitative level it shows how MB absorbance works as a good DO indicator.
(0.10 MB PDF)

Figure S4 Bacteriostasis and phenotypic drug resistance of AA-treated M. tb strains. (A) Bacteriostasis of AA-treated strains. (B) Comparison of control vs. treated M. tb cultures post-INH treatment. Cultures were exposed to 10 mM AA for 1 day and treated with INH for 4 days in the presence of AA. 100% represents CFU of ‘no drug culture’; INH represents CFU of surviving bacteria after drug treatment, both on day 5. Mean ± SD of three independent cultures is shown. The differences in CFUs obtained with various strains that were treated or not treated with AA was statistically significant on day 2 (***, p<0.001).
(0.75 MB DOC)

Figure S5 Growth and INH sensitivity of DETA/NO-treated M. tb strains. Growth and viability of DETA/NO-treated M.tb strains monitored by CFU counts after exposure to 50 µM DETA/NO for 1 day followed by a 4-day INH drug treatment. Data is shown from one of two independent experiments with similar results.
(0.03 MB PDF)
