# Supplementary material for: Mycobacterium tuberculosis Transcriptional Adaptation, Growth Arrest and Dormancy Phenotype Development Is Triggered by Vitamin C
Source: PLoS One. 2010 May 27;5(5):e10860. doi: 10.1371/journal.pone.0010860 (PMC2877710; doi:10.1371/journal.pone.0010860)
Supplement: Text S1 — Some reactions of Ascorbic acid. (0.07 MB DOC) [file pone.0010860.s001.doc]

**Text S1**

**Some reactions of Ascorbic acid**

AA participates in a large number of reactions due to its chemical reactivity. The reactions that are relevant to the action of AA observed in this study are given below.

1. O_2_ scavenging action. AA scavenges O_2_ (1) and leads to the generation of H_2_O_2_ .

AA + O_2_ → DHA + H_2_O_2_

1. Iron reducing effect of AA. AA is used to maintain iron in the ferrous state in biological assays (2).

4Fe^3+^ + 2AA + O_2_ → 2DHA + 4Fe^2+^ + 2H_2_O

3. Reaction of methylene blue with AA (3).

Methylene blue + AA → Leucomethylene blue + DHA

(blue) (colourless)

References.

1. Scarpa M, Stevanato R, Viglino P, Rigo A (1983) Superoxide ion as active intermediate in the autoxidation of ascorbate by molecular oxygen. Effect of superoxide dismutase. J Biol Chem 258: 6695-6697.
2. Agranoff D, Monahan IM, Mangan JA, Butcher PD, Krishna S (1999) *Mycobacterium tuberculosis* expresses a novel pH-dependent divalent cation transporter belonging to the Nramp family. J Exp Med 190: 717-724.
3. Mowry S, Ogren PJ (1999) Kinetics of Methylene Blue Reduction by Ascorbic Acid. *J.* Chem. Educ. 76: 970-973.
