# Supplementary material for: Mycobacterium tuberculosis Transcriptional Adaptation, Growth Arrest and Dormancy Phenotype Development Is Triggered by Vitamin C
Source: PLoS One. 2010 May 27;5(5):e10860. doi: 10.1371/journal.pone.0010860 (PMC2877710; doi:10.1371/journal.pone.0010860)
Supplement: Table S1 — Plasmids used in this study. (0.03 MB DOC) [file pone.0010860.s002.doc]

**Table S1. Plasmids used in this study**

| **Plasmid** | **Relevant Feature(s)^*^** | **Source or reference** |
| --- | --- | --- |
| **pFPVH** | *E. coli*-mycobacterial shuttle plasmid with promoterless *gfp*, Hyg^r^ | D. K. Saini and J. S. Tyagi,  unpublished data |
| **p3134c-1** | pFPV27 containing *Rv3134c* promoter (−608 to +90), Hyg^r^ | Chauhan and Tyagi, 2008a |
| **p1738** | pFPV27 containing *Rv1738* promoter (−203 to +74); Hyg^r^ | Chauhan and Tyagi, 2008b |

^*^ The coordinates of the promoters (in parentheses) are with reference to the translational start site for *Rv3134c* promoter; and transcriptional start site for the *Rv1738* promoter.

Hyg^r^, hygromycin resistance.

References.

Chauhan, S., and J. S. Tyagi. 2008a. Cooperative binding of phosphorylated DevR to upstream sites is necessary and sufficient for activation of the Rv3134c-devRS operon in Mycobacterium tuberculosis: implication in the induction of DevR target genes. J Bacteriol 190:4301-12.

Chauhan, S., and J. S. Tyagi. 2008b. Interaction of DevR with multiple binding sites synergistically activates divergent transcription of narK2-Rv1738 genes in Mycobacterium tuberculosis. J Bacteriol 190:5394-403.
