# Supplementary material for: Mycobacterium tuberculosis Transcriptional Adaptation, Growth Arrest and Dormancy Phenotype Development Is Triggered by Vitamin C
Source: PLoS One. 2010 May 27;5(5):e10860. doi: 10.1371/journal.pone.0010860 (PMC2877710; doi:10.1371/journal.pone.0010860)
Supplement: Table S2 — Primers used for quantitative reverse transcriptase-PCR analysis. (0.04 MB PDF) [file pone.0010860.s003.pdf]

Table S2. Primers used for quantitative reverse transcriptase-PCR analysis

Gene (Rv No.)	Primer*	Primer sequence 5’ ⟶ 3’
16S rRNA/rrs	*16S rRNA* F	ATGACGGCCTTCGGGTTGTAA
	16S rRNA R	CGGCTGCTGGCACGTAGTTG
devR (*Rv3133c*)	*devR* f4	CCGATCTGCGCTGTCTGATC
	devR r3	GTCCAGCGCCCACATCTTT
devS (*Rv3132c*)	*devSint1* F	TACTGACCGACCGGGATCGT
	devSint1 R	AGAGCCGCTGGATGACATGG
Rv3134c	*Rv3134c* F	CTGGCTGGGTCGGCCTTA
	Rv3134c R	GCTGACCTGGGAGGTTGTCG
hspX (*Rv2031c*)	*Rv2031c* F	CGCACCGAGCAGAAGGA
	Rv2031c R	ACCGTGCGAACGAAGGAA
fdxA (*Rv2007c*)	*fdxA* F	TGTCCGGTCGACTGTATCTATGA
	fdxA R	GGCAGGCCGGTTTGC
icl (*Rv0467*)	*icl* F	GGACCAGATGCTGGCCTA
	icl R	CTGCCAGCTCCTTCTGGA
espA (*Rv3616c*)	*Rv3616c* F	CCTCGGAGAAGTGTGGGAGT
	Rv3616c R	CGAGAGAACAGTCCGGTCAC
mymA (*Rv3083()*	*Rv3083* F	CGATCCCCGTCGTACATC
	Rv3083 R	ACTCGTAGACCCAGCGATCC
fadD13 (*Rv3089*)	*Rv3089* F	GCCCGACTTCCGCTACTT
	Rv3089 R	CGTAACCCTGCACGACCT
ahpC (*Rv2428*)	*ahpC* F	CCGGCGACTACTTCACCA
	ahpC R	TCGGTAGGGCACACGAAC
furA (*Rv1909c*)	*furA* F	GCCGATGTCGACTGTGCT
	furA R	GTAGATGACCTCCGCCTCGT
katG (*Rv1908c*)	*katG*F	CCTCCGAGTCACTGACCAAC
	*katG*R	GGTAGGTCCCGTCATCTGCT
glbN (*Rv1542c*)	*Rv1542c* F	GCCCTCCGAAACGATCAC
	Rv1542c R	TCAGACTGGTGCCGTGGT
dosT (*Rv2027c*)	*Rv2027c* F	CGACCTGGTCATCGTAGAGG
	Rv2027c R	AAGACTCCCCCGATCGAC
Rv1771	*Rv1771* F	GGGGAAGTGCTCAGTCTGTC
	Rv1771 R	GAACCGTCTGCAGGGTGA

*F and R suffix in each primer name refer to forward and reverse primer, respectively.
