# Supplementary figures and images for: Mycobacterium tuberculosis Transcriptional Adaptation, Growth Arrest and Dormancy Phenotype Development Is Triggered by Vitamin C
Source: PLoS One. 2010 May 27;5(5):e10860. doi: 10.1371/journal.pone.0010860 (PMC2877710; doi:10.1371/journal.pone.0010860)

Figure S3

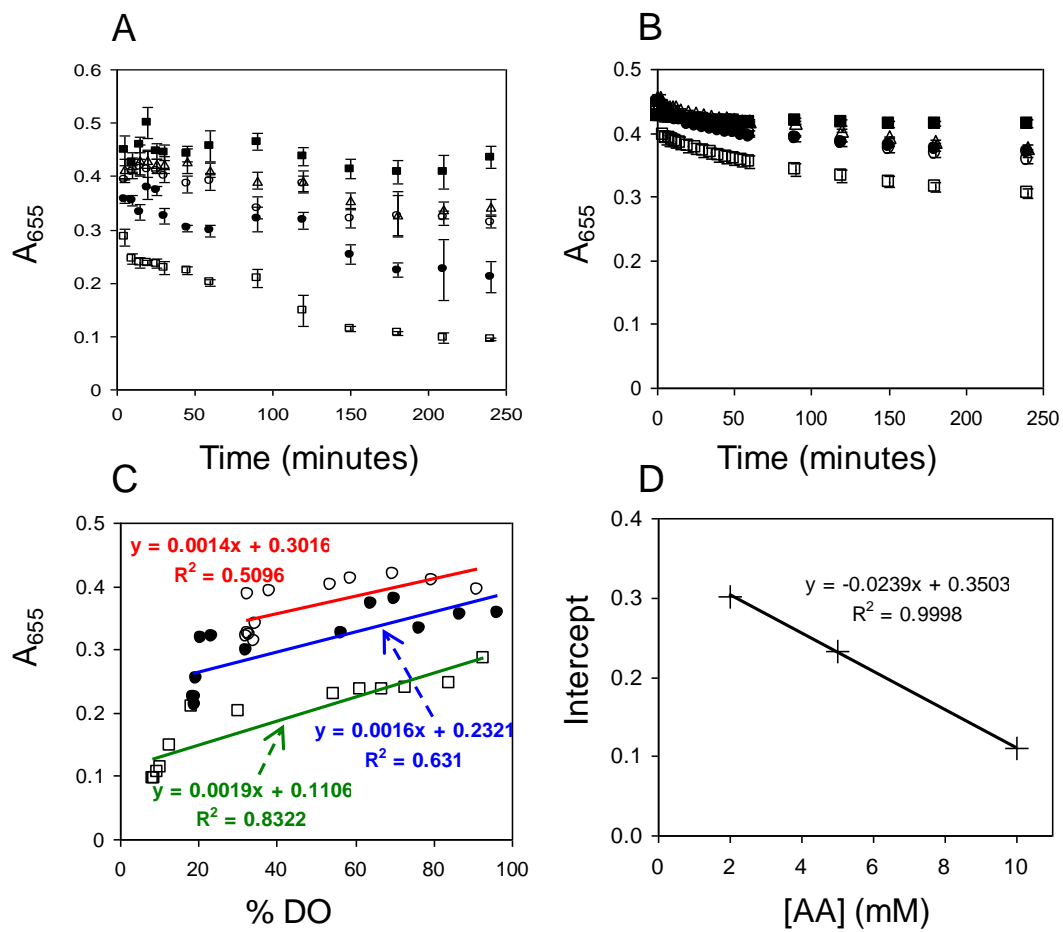

Supplement: Figure S3 — Methylene blue (MB) absorbance as a function of dissolved oxygen. (A) MB absorbance (A655) in culture medium under shaking conditions (without cells) decreases with time for AA concentrations of 0 (▪), 1 mM (Δ), 2 mM (○), 5 mM (•), 10 mM (□). (B) DHA does not lead to a substantial decolorization of MB (concentrations of DHA used and symbols are same as those in (a)). (C) A655 plotted as a function of DO can be approximated as a linear relationship (regression coefficients of 0.51, 0.63, 0.82 resp.) of the form A655 = (1.63×10^−3^±2.52×10^−4^)DO + Intercept (see below), where DO is expressed in %DO, for AA concentrations of 2 mM (○), 5 mM (•), 10 mM (□). (D) The intercepts from (c) show a linear inverse dependence on [AA] of the form Intercept = −0.0239[AA] + 0.3503, where [AA] is in mM. The calibration of MB absorbance as a function of dissolved O2 can be simplistically approximated as A655∼0.0016(DO) + (−0.0239[AA] + 0.3503), where DO is expressed as %DO and [AA] is in mM. While the linear relationship is certainly a simplified approximation, at least on a semi-quantitative level it shows how MB absorbance works as a good DO indicator. (0.10 MB PDF) [file pone.0010860.s006.pdf]
