# Supplementary material for: Mycobacterium tuberculosis Transcriptional Adaptation, Growth Arrest and Dormancy Phenotype Development Is Triggered by Vitamin C
Source: PLoS One. 2010 May 27;5(5):e10860. doi: 10.1371/journal.pone.0010860 (PMC2877710; doi:10.1371/journal.pone.0010860)
Supplement: Figure S4 — Bacteriostasis and phenotypic drug resistance of AA-treated M. tb strains. (A) Bacteriostasis of AA-treated strains. (B) Comparison of control vs. treated M. tb cultures post-INH treatment. Cultures were exposed to 10 mM AA for 1 day and treated with INH for 4 days in the presence of AA. 100% represents CFU of ‘no drug culture’; INH represents CFU of surviving bacteria after drug treatment, both on day 5. Mean ± SD of three independent cultures is shown. The differences in CFUs obtained with various strains that were treated or not treated with AA was statistically significant on day 2 (***, p<0.001). (0.75 MB DOC) [file pone.0010860.s007.doc]

**Figure S4**
