# Supplementary material for: Mycobacterium tuberculosis Transcriptional Adaptation, Growth Arrest and Dormancy Phenotype Development Is Triggered by Vitamin C
Source: PLoS One. 2010 May 27;5(5):e10860. doi: 10.1371/journal.pone.0010860 (PMC2877710; doi:10.1371/journal.pone.0010860)
Supplement: Figure S5 — Growth and INH sensitivity of DETA/NO-treated M. tb strains. Growth and viability of DETA/NO-treated M.tb strains monitored by CFU counts after exposure to 50 µM DETA/NO for 1 day followed by a 4-day INH drug treatment. Data is shown from one of two independent experiments with similar results. (0.03 MB PDF) [file pone.0010860.s008.pdf]

Figure S5

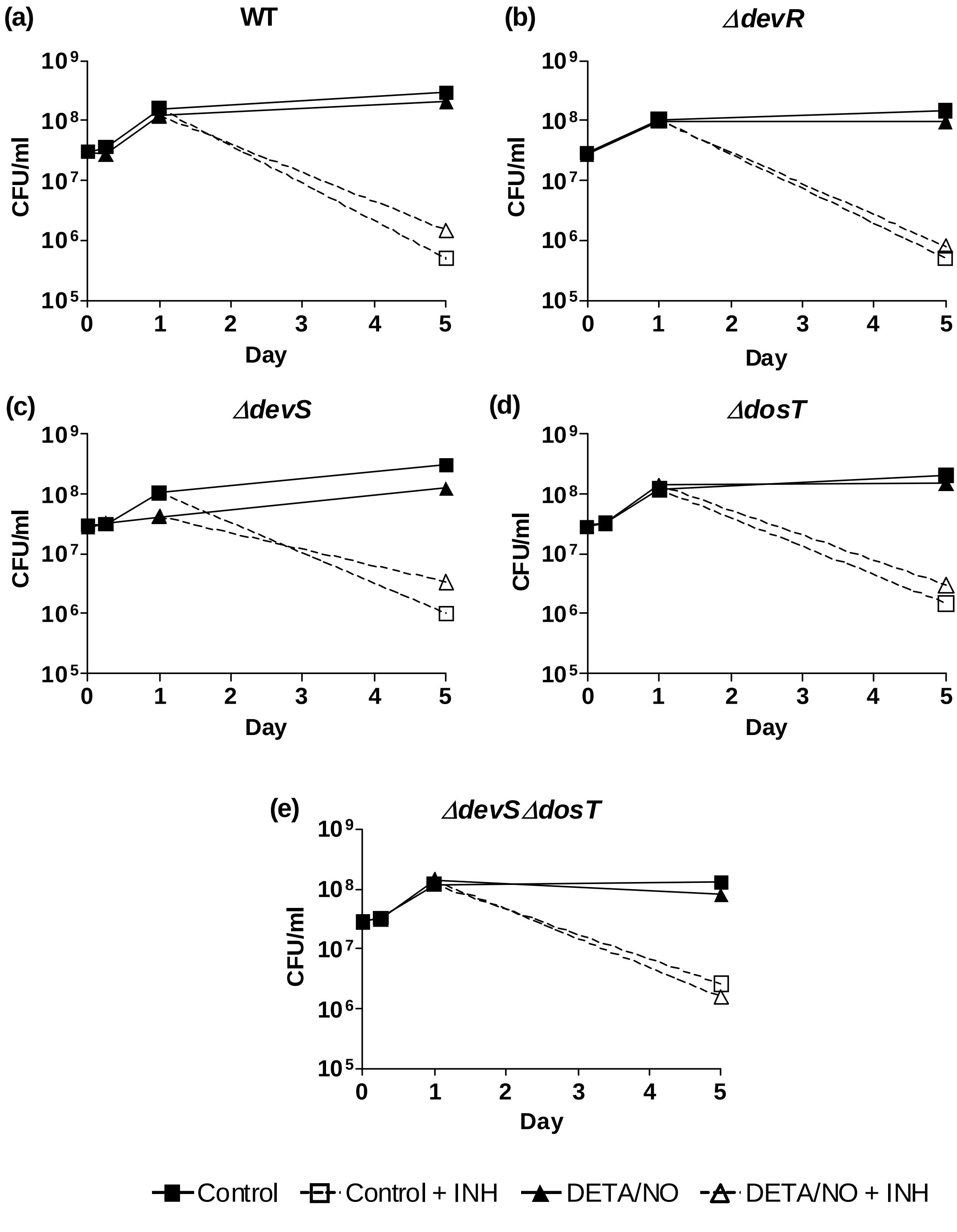

<u>Figure S5.</u> Growth and INH sensitivity of DETA/NO-treated *M. tb* strains. Growth and viability of DETA/NO-treated *M.tb* strains monitored by CFU counts after exposure to 50 µM DETA/NO for 1 day followed by a 4-day INH drug treatment. Data is shown from one of two independent experiments with similar results.
